# The impact of heavy nurse workload and patient/family complaints on workplace violence: An application of human factors framework

**DOI:** 10.1002/nop2.444

**Published:** 2020-01-15

**Authors:** Farinaz Havaei, Maura MacPhee

**Affiliations:** ^1^ School of Nursing University of British Columbia Vancouver BC Canada

**Keywords:** human factors framework, nurses, patient and family complaints, workload, workplace violence

## Abstract

**Aim:**

To examine the relationships between workload factors at different systems levels (unit level, job level and task level), patients/family complaints and nurse reports of patient violence towards them using a human factors framework.

**Design:**

This is a secondary analysis of cross‐sectional data.

**Methods:**

Data from 528 nurses working in medical–surgical settings in British Columbia, Canada, were analysed. At the unit‐level workload factors included patient‐RN ratios, patient acuity and dependency; at the job‐level perceptions of heavy workload, undone nursing tasks and compromised professional standards due to workload; and at the task‐level interruptions to workflow.

**Results:**

Workload factors at multiple levels were directly related to workplace violence. Nurses' increased reports of compromised standards (job level) and interruptions (task level) were related to increased reports of physical and emotional violence, and higher patient acuity (unit level) was related to increased reports of emotional violence. Patient/family complaints mediated the relationship between almost all the workload factors and workplace violence.

## 1 INTRODUCTION

The prevalence of workplace violence in the healthcare sector continues to rise globally. Nurses are particularly at risk for workplace violence due to the nature of their work (Canadian Federation of Nurses' Union [CFNU], 2017). Patients and their families/visitors are known to be the most common perpetrators of violence towards nurses (Spector, Zhou, & Che, 2014). Although there is extensive literature about the relationships between work environment factors and nurse outcomes (Aiken, Sloane, Bruyneel, Van den Heede, & Sermeus, 2013; Aiken et al., 2011; Kutney‐Lee et al., 2015; Kutney‐Lee, Wu, Sloane, & Aiken, 2013; McHugh & Ma, 2014), very few studies have examined how work environment factors, such as workload, contribute to violence towards nurses. We know that when nurses are overworked, there is a rise in workplace violence (CFNU, 2017; Casey, 2019); and nurses believe that heavy workloads influence their capacity to deliver care effectively, resulting in anxious and frustrated patients who become violent (Pich, Kable, & Hazelton, 2017; Shields & Wilkins, 2009). Because patients/families are the main perpetrators of violence towards nurses, this study investigated whether patient/family complaints about nursing care delivery can serve as an indicator of patient violence towards nurses. In particular, the purpose of this study was to examine the relationships between workload factors at different systems levels, patients/family complaints and nurse reports of patient violence towards them.

## 2 BACKGROUND

In one national Canadian study, when nurses reported inadequate staffing and resources in their work environments, they also reported more frequent exposure to physical and emotional workplace violence perpetuated by patients, even after controlling for nurse and job characteristics (Shields & Wilkins, 2009). A longitudinal cohort study of more than 34,000 nurses across eight European countries found heavy workload, operationalized as time pressure, was associated with a higher likelihood of violence from patients and harassment by other organizational employees (Camerino, Estryn‐Behar, Conway, van Der, & Hasselhorn, 2008). A cross‐sectional survey study of about 2,500 Australian nurses examined the effect of several workload factors on their exposure to workplace violence—operationalized as physical violence, emotional violence and threat of violence perpetuated by patients, families/visitors and organizational employees (Roche, Diers, Duffield, & Catling‐Paull, 2010). Unanticipated changes in patient acuity were associated with a higher likelihood of threat of violence; higher RN staffing levels were associated with a lower likelihood of threat of violence and physical violence; and more undone nursing tasks were associated with physical, emotional and threat of violence (Roche et al., 2010). Although this study focused on various sources of workplace violence, patients by far were the most common perpetuators of all three types of workplace violence. More recently, Pich et al. (2017) found that nurses who had experienced verbal and physical workplace violence from patients in the last 6 months were two times more likely to have experienced heavy workload and time management issues. In this study, nurses reported workload, time management issues and inadequate staffing as the most common causes of workplace violence perpetuated by patients. Similarly, a descriptive study of 174 Jordanian emergency nurses reported workload was the most common cause of workplace violence, two times more than caring for patients with dementia or Alzheimer's disease (Darawad, Al‐Hussami, Saleh, Mustafa, & Odeh, 2015).

The research evidence suggests an association between nurses' heavy workloads and negative patient outcomes, including patient complaints and dissatisfaction. For example, some studies showed that heavy nurses' workloads, operationalized as nurse staffing levels and higher number of undone nursing tasks, were associated with more frequent patient/family complaints (Aiken et al., 2010; Thomas‐Hawkins, Flynn, & Clarke, 2008). Others found a relationship between heavy nurse workload and higher patient dissatisfaction (Kutney‐Lee et al., 2009). Recently, the Canadian Centre for Occupational Health and Safety (2019) identified clients' complaints of unfair treatment as an important warning sign of workplace violence. To our knowledge, no previous research has examined the association between patient/family complaints and workplace violence. In addition, the small body of research that examined associations between nurses' workload, patient complaints and violence towards nurses focused predominantly on unit‐level workload factors.

### 2.1 Theoretical framework

Workload is a complex construct requiring considerations of multiple factors at multiple levels (Carayon & Gurses, 2008; Holden et al., 2011). Despite this complexity, a majority of workplace violence research has examined workload factors at one systems level (e.g. unit level) without using a systematic theoretical framework. To address this limitation, the conceptualization of workload is informed by a human factors framework in this study (Holden et al., 2011; MacPhee, Dahinten, & Havaei, 2017). According to this framework, an interaction between demands and resources produces workload at multiple levels including unit level, job level and task level. Holden et al. (2011) conceptualized unit‐level workload as staffing considerations; job‐level workload as the general and specific demands of the job including the amount of work and the level of concentration required to complete it in a given day; and task‐level workload as the demands (e.g. need for multitasking) and resources (e.g. technology) for a specific nursing task such as medication administration. Holden et al. (2011) validated their framework among nurses. Heavy workload at the three levels was associated with higher job dissatisfaction and burnout, but only job‐level and task‐level workload factors were associated with more frequent medication errors (Holden et al., 2011). Overall, this research showed that nurse and patient outcomes may vary, depending on the level of workload (i.e. unit, job, task).

MacPhee et al. (2017) used the Holden et al. human factors framework to conceptualize workload and examine its impact on patient and nurse outcomes. In this study, unit‐level workload factors included nurse reported patient‐RN ratios, patient acuity and patient dependency; job‐level workload factors included nurses' perceptions of heavy workload, nursing tasks left undone and compromised professionals standards due to workload; and task‐level workload factors included the frequency of interruptions to workflow. The authors found heavy workload at all three levels was associated with adverse patient outcomes, such as patient falls and urinary tract infections, and negative nurse outcomes, such as emotional exhaustion (MacPhee et al., 2017).

### 2.2 Research question

In this study, the MacPhee et al. (2017) workload framework and operational definitions were used to examine the following research questions:

1. Are there any relationships between workload factors and physical and emotional workplace violence after accounting for nurse characteristics?
2. Are there any relationships between patient/family complaints and physical and emotional workplace violence after accounting for nurse characteristics?
3. Do patient/family complaints mediate the relationship between workload factors and physical and emotional workplace violence?

## 3 THE STUDY

### 3.1 Design

This study was a secondary analysis of cross‐sectional survey data from 528 nurses working in medical and/or surgical settings in British Columbia (BC), Canada.

### 3.2 Method

#### 3.2.1 Sample

In the larger study, a proportionate stratified random sample of registered nurses (RNs) and licensed practical nurses (LPNs), based on geographic regions (health authority) and employment status (full‐time, part‐time and causal), was invited to participate in the study in 2015 (blinded). A total of 1,876 acute care nurses in direct care, leadership and educator roles participated. For this secondary analysis, the inclusion criteria consisted of direct care nurses from medical, surgical or medical–surgical areas, resulting in a sample of 528 nurses.

#### 3.2.2 Data collection

In the larger study, data were collected using an electronic survey platform. The study survey consisted of a series of researched developed questions and validated items and scales. For this study, key predictors include workload factors at the unit level, job level and task level operationalized based on MacPhee et al. (2017); patient/family complaints; and nurse demographics. Workplace violence was the key outcome variable.

##### Workload factors

###### Unit‐level RN Staffing Levels

A ratio of patients per RN was obtained based on two questions that asked nurses to identify the number of patients and the number of direct care RNs on their unit over the last shift. These questions were previously validated in the international RN4CAST research (Aiken et al., 2013; Sermeus et al., 2011).

###### Unit‐level patient acuity and dependency

Two items based on the American Association of Critical Care Nurses' Synergy Model were used to assess patient acuity and dependency (Curley, 2007). While patient acuity refers to the instability, complexity and unpredictability of patients' condition, patient dependency describes patient's ability to perform their own activities of daily living. These definitions were provided on the survey and nurses were asked to rate the average acuity (1 = not at all acute, 4 = very acute) and dependency (very independent, 4 = very dependent) of their patients during the last month. For this analysis, acuity and dependency scores were dichotomized into 0 = not at all or somewhat acute, 1 = moderately or very acute; and 0 = very or somewhat independent, 1 = somewhat or very dependent. These questions were previously validated by MacPhee et al. (2017).

###### Job‐level heavy workload

Three workload items based on the Canadian National Survey on the Work and Health of Nurses were used to assess the general amount of work nurses have to complete (Statistics Canada, 2006). The items asked about the frequency by which nurses arrived early or stayed late, worked through breaks to complete work and felt they had to complete “too much work” during last the year. Mean scores were obtained with higher scores indicating more job‐level nurse workload. A principal component analysis with varimax rotation extracted a unidimensional factor explaining 69.1% of the variance with factor loading ranging from 0.65–0.74. Cronbach's alpha was 0.78 suggesting satisfactory internal consistency.

###### Job‐level undone nursing tasks

One question based on a study conducted by Ball and colleagues (Ball, Murrells, Rafferty, Morrow, & Griffiths, 2014) was used to identify the number of nursing tasks undone over the last shift. The question showed a list of 14 nursing activities such as on‐time medication administration and skin care. Nurses were asked to select all the activities that were necessary but left undone during their most recent shift due to lack of time. MacPhee et al. (2017) further validated this question among a nursing sample.

###### Job‐level compromised professional standards

A single researcher developed question was used to identify the frequency of compromised professional nursing standards over the last year due to workload. Response options were rated on a seven‐point scale from 0 (never) to 6 (everyday). This question was validated using nurse focus groups as well as in a study by MacPhee et al. (2017).

###### Task‐level interruptions to workflow

Three researcher developed items, based on a focused literature review, asked nurses about the frequency of being interrupted over the last month during patient treatments, documentation and patient handover. Response options were on a seven‐point scale of 0 (never) to 6 (everyday). Mean scores were obtained with higher scores indicating higher frequency of interruptions. Nurse focus groups were used to content validate these questions (MacPhee et al., 2017). Also, a principal component analysis with varimax rotation extracted a unidimensional factor explaining 74.6% of the variance with factor loading ranging from 0.59–0.84. Cronbach's alpha was 0.81 suggesting good internal consistency.

##### Patient/family complaints

A single validated question, based on RN4CAST research, was used to identify the frequency by which nurses received complaints from their patients and their families over the last year (Aiken et al., 2013). Response options ranged from never (0) to every day (6).

##### Demographics

A series of questions were used to assess respondents' age, gender, professional designation (RN vs. LPN), highest nursing education (diploma vs. degree), years of nursing experience and employment status (full‐time vs. part‐time or casual).

##### Workplace violence

###### Emotional and physical abuse

Two workplace violence items were adapted from the 2005 National Survey of the Work and Health of Nurses (Statistics Canada, 2006) and the study by Hesketh and colleagues (Hesketh et al., 2003). The questions asked participants to identify the frequency by which they had experienced emotional and physical abuse from patients and/or families in their primary workplace over the past year (0 = never, 6 = everyday). Hesketh et al. (2003) established the convergent validity of the items by linking more frequent workplace violence exposure to higher job dissatisfaction of Canadian nurses.

#### 3.2.3 Analysis

Key methods of data analysis were descriptive statistics, bivariate correlations and hierarchical multiple regression using the Statistical Package for Social Sciences for Windows 25.0 (SPSS Inc.). In particular, the first and second research questions were examined using hierarchical multiple regression; nurse characteristics were entered into the first regression model followed by unit‐level, job‐level and task‐level workload factors in the second, third and fourth models, respectively, followed by patient/family complaints in the fifth model. To save power, only variables that showed significant bivariate correlations with one or both of the outcome variables were included in the regression model. To further save power, demographic variables that were not related to workplace violence in the regression model were dropped from the model. The third research question was examined using Baron and Kenny's recommendations (1986) and Preacher and Leonardelli's Sobel test (2010). According to Baron and Kenny, mediation is dependent on three to four conditions: (a) a significant beta coefficient when the independent variable is regressed on the outcome variable; (b) a significant beta coefficient when the independent variable is regressed on the mediator; (c) a significant beta coefficient of the mediator regressed on the outcome variable controlling for the independent variable; and (d) non‐significant beta coefficient of independent variable regressed on outcome variable controlling for the mediator. While all four conditions are required to establish full mediation, partial mediation requires only the first three conditions.

#### 3.2.4 Ethics

Participants were informed that survey completion and submission would indicate consent to be included in the study. Ethics approval was obtained from the university behavioural ethics review board (Approval number: H14‐00789).

## 4 RESULTS

Table 1 presents the demographic characteristics of 528 nurses with an average age of 39 years and 10 years of nursing experience. Among the overwhelmingly female sample, three‐quarters were RNs. More than half of the sample had a nursing degree and a full‐time position.

**Table 1:** Descriptive statistics of key study variables (*N* = 528)

	*f* (%)	*M* (*SD*)
Age	–	39.4 (11.8)
Gender		
Male	26 (5.0%)	**–**
Female	497 (95.0%)	**–**
Professional designation		
Licensed practical nurse (LPN)	128 (24.2%)	–
Registered nurse (RN)	400 (75.8%)	–
Education		
Diploma or certificate	220 (41.7%)	–
Baccalaureate or masters	308 (58.3)	–
Experience	‐	10.2 (9.6)
Employment status		
Full‐time	292 (55.3%)	–
Part‐time or casual	236 (44.7%)	–
Patient‐RN ratio	–	7.0 (4.5)
Patient acuity		
Not at all or somewhat acute	129 (24.4%)	–
Moderately or very acute	399 (75.6%)	–
Patient dependency		
Very or somewhat independent	79 (15.0%)	–
Very or somewhat dependent	447 (85%)	–
Heavy workload	–	4.1 (1.4)
Undone tasks	–	4.6 (3.1)
Compromised standards	–	3.4 (2.0)
Interruptions	–	4.8 (1.2)
Patient/family complaints	–	2.4 (1.7)
Physical violence	–	1.8 (1.6)
Emotional violence	–	2.4 (1.7)

Table 1 also shows the descriptive statistics on other study variables. At the unit level, nurses reported an average of 7 patients per RN; and more than three‐quarters of them classified their patients as moderately or very acute; and very or somewhat dependent. At the job level, on average, nurses experienced heavy workload once a week; left 5 necessary nursing tasks undone; and compromised professional standards a few times a month. At the task level, on average, nurses were interrupted a few times a week. Nurses were exposed to physical and emotional violence an average of once a month.

Table 2 presents the bivariate correlations. With the exception of age, nursing experience and patient dependency, other variables were significantly related to physical and/or emotional violence with correlations ranging from 0.10–0.57. Non‐significant variables were not included in our regression analyses, and gender was excluded because of the small proportion of male nurses in the sample (only 5%).

**Table 2:** Bivariate correlations between study variables (*N* = 528)

	2	3	4	5	6	7	8	9	10	11	12	13	14	15	16
1. Age	−0.05	−0.20**	−0.58**	0.80**	−0.17**	0.06	−0.05	−0.10*	−0.04	−0.02	−0.01	0.04	0.07	0.04	0.03
2. Gender^a^	–	0.18**	0.15**	0.04	−0.03	0.05	0.08	0.08	−0.09*	−0.10*	−0.06	0.00	−0.04	−0.10*	−0.07
3. Designation^b^		–	0.67**	−0.02	−0.03	−0.16**	0.21**	0.03	0.06	−0.07	−0.03	0.00	−0.08	−0.16**	−0.08
4. Education^c^			–	−0.47**	0.01	−0.12**	0.15**	0.05	0.05	−0.03	−0.05	−0.03	−0.07	−0.12**	−0.08
5. Experience				–	−0.14**	0.03	0.00	−0.11*	−0.06	−0.07	−0.05	0.06	0.10*	0.00	−0.01
6. Employment status^d^					–	−0.03	−0.08	−0.04	−0.08	0.01	−0.04	−0.18**	−0.10*	−0.19**	−0.17**
7. Patient‐RN ratios						–	−0.10*	0.03	0.04	0.14**	0.12**	0.01	0.18**	0.14**	0.11*
8. Patient acuity^e^							–	0.07	0.21**	0.12**	0.12**	0.30**	0.13**	0.12**	0.18**
9. Patient dependency^f^								–	0.12**	0.16**	0.05	0.15**	0.03	0.01	0.06
10. Heavy workload									–	0.43**	0.51**	0.46**	0.23**	0.23**	0.32**
11. Undone tasks										–	0.44**	0.35**	0.33**	0.28**	0.28**
12. Compromised standards											–	0.39**	0.28**	0.31**	0.36**
13. Interruptions												–	0.33**	0.31**	0.35**
14. Patient/family complaints													–	0.33**	0.42**
15. Physical violence														–	0.57**
16. Emotional violence															–

a (0 = male, 1 = female).

b (0 = LPN, 1 = RN).

c (0 = diploma, 1 = degree).

d (0 = full‐time, 1 = part‐time or casual).

e (0 = not at all or somewhat acute, 1 = moderately or very acute).

f (0 = very or somewhat independent, 1 = somewhat or very dependent).

* *p* < .05.

** *p* < .01.

*** *p* < .001.

### 4.1 Research questions 1 and 2

Table 3 presents the hierarchical multiple regression analysis for physical violence. After accounting for demographic characteristics, unit‐level patient‐RN ratios and patient acuity were significantly related to physical violence in model 2, but after the addition of job‐level and task‐level workload factors in model 4, patient acuities ceased to be statistically significant. In model 4, in addition to professional designation (*β *= −0.14, *p *< .05) and employment status (*β *= −0.15, *p *< .05), workload factors including unit‐level patient‐RN ratios (*β *= 0.08, *p *< .05), job‐level undone tasks (*β *= 0.12, *p *< .01) and compromised standards (*β *= 0.16, *p *< .01) and task‐level interruptions (*β *= 0.15, *p *< .01) were significantly related to physical violence. After the introduction of patient/family complaints in model 5, unit‐level patient‐RN ratios and job‐level undone tasks ceased to be statistically significant. In model 5, patient/family complaints (*β *= 0.18, *p *< .001) and employment status (*β *= −0.15, *p *< .001) were the strongest predictors of physical violence (*F *(9, 518) = 17.30, *p *< .001).

**Table 3:** Results of hierarchical multiple regression analyses for physical workplace violence from patients and/or visitors (*N* = 528)

Predictor variables	Mode 1 β (95% CI)	Model 2 β (95% CI)	Model 3 β (95% CI)	Model 4 β (95% CI)	Model 5 β (95% CI)
Professional designation^a^	−0.17*** (−0.92, −0.31)	−0.18*** (−0.97, −0.35)	−0.16*** (−0.89, −0.30)	−0.15*** (−0.86, −0.27)	−0.14*** (−0.82, −0.24)
Employment status^b^	−0.20 *** (−0.88, −0.36)	−0.18*** (−0.83, −0.31)	−0.18*** (−0.80, −0.31)	−0.15*** (−0.74, −0.24)	−0.15*** (−0.70, −0.21)
Patient‐RN ratios		0.12** (0.01, 0.07)	0.08 (−0.00, 0.05)	0.08* (0.00, 0.06)	0.06 (−0.01, 0.05)
Patient acuities^c^		0.15*** (0.24, 0.86)	0.09* (0.04, 0.64)	0.06 (−0.07, 0.54)	0.05 (−0.11, 0.49)
Heavy workload			0.05 (−0.05, 0.17)	0.01 (−0.10, 0.12)	0.01 (−0.10, 0.12)
Undone tasks			0.14** (0.03, 0.12)	0.12** (0.02, 0.11)	0.09 (−0.0, 0.10)
Compromised standards			0.19*** (0.07, 0.22)	0.16** (0.05, 0.20)	0.14** (0.04, 0.18)
Interruptions				0.15** (0.07, 0.32)	0.11* (0.02, 0.27)
Patient/Family complaints					0.18*** (0.09, 0.24)
Change in *R^2^*	6.5%***	3.4%***	9.1%***	1.5%**	2.7%***
*R^2^*	6.5%***	9.8%***	18.9***	20.4%**	23.1%***

Note

*F *(9, 518) = 17.30, *p *< .001.

a (0 = LPN, 1 = RN).

b (0 = full‐time, 1 = part‐time or casual).

c (0 = not at all or somewhat acute, 1 = moderately or very acute).

* *p* < .05.

** *p* < .01.

*** *p* < .001.

Table 4 presents the hierarchical multiple regression analysis for emotional violence. After accounting for demographic characteristics, unit‐level patient‐RN ratios and patient acuity were significantly related to emotional violence in model 2, but after the addition of job‐level and task‐level workload factors in model 4, patient‐RN ratios ceased to be statistically significant. In model 4, in addition to professional designation (*β *= −0.09, *p *< .05) and employment status (*β *= −0.12, *p *< .01), workload factors including unit‐level patient acuities (*β *= 0.11, *p *< .05), job‐level compromised standards (*β *= 0.19, *p *< .001) and task‐level interruptions (*β *= 0.15, *p *< .01) were significantly related to emotional violence. After the introduction of patient/family complaints in model 5, these variables remained statistically significant. In model 5, patient/family complaints (*β *= 0.28, *p *< .001) and job‐level compromised standards (*β *= 0.16, *p *< .01) were the strongest predictors of emotional violence (*F *(9, 518) = 23.11, *p *< .001).

**Table 4:** Results of hierarchical multiple regression analyses for emotional workplace violence from patients and/or visitors (*N* = 528)

Predictor variables	Mode 1 β (95% CI)	Model 2 β (95% CI)	Model 3 β (95% CI)	Model 4 β (95% CI)	Model 5 β (95% CI)
Professional designation^a^	−0.09* (−0.69, −0.01)	−0.11** (−0.80, −0.11)	−0.10* (−0.73, −0.09)	−0.09* (−0.69, −0.06)	−0.08* (−0.62, −0.00)
Employment status^b^	−0.17*** (−0.88, −0.30)	−0.15*** (−0.81, −0.24)	−0.14*** (−0.75, −0.22)	−0.12** (−0.68, −0.14)	−0.10** (−0.62, −0.10)
Patient‐RN ratios		0.11* (0.01, 0.07)	0.06 (−0.01, 0.05)	0.06 (−0.01, 0.06)	0.03 (−0.02, 0.04)
Patient acuities^c^		0.21*** (0.49, 1.17)	0.14** (0.22, 0.87)	0.11* (0.09, 0.75)	0.09* (0.04, 0.67)
Heavy workload			0.13** (0.05, 0.28)	0.09 (−0.01, 0.24)	0.09 (−0.00, 0.23)
Undone tasks			0.10* (0.01, 0.10)	0.08 (−0.01, 0.09)	0.03 (−0.03, 0.06)
Compromised standards			0.21*** (0.10, 0.26)	0.19*** (0.08, 0.24)	0.16** (0.06, 0.21)
Interruptions				0.15** (0.09, 0.35)	0.09* (0.01, 0.26)
Patient/Family complaints					0.28*** (0.20, 0.36)
Change in *R^2^*	3.6%***	4.9%***	12.3%***	1.6%**	6.4%***
*R^2^*	3.6%***	8.4%***	20.7%***	22.3%**	28.6%***

Note

*F *(9, 518) = 23.11, *p *< .001.

a (0 = LPN, 1 = RN).

b (0 = full‐time, 1 = part‐time or casual).

c (0 = not at all or somewhat acute, 1 = moderately or very acute).

* *p* < .05.

** *p* < .01.

*** *p* < .001.

The positive beta associated with job‐level compromised standards and task‐level interruptions suggests that when nurses compromised their professional standards due to workload and were interrupted during their work more frequently, they reported more frequent exposure to physical and emotional violence. Similarly, the positive beta associated with patient/family complaints suggests that more frequent complaints from patients and/or their families were associated with more frequent exposure to physical and emotional violence. The negative betas associated with professional designation and employment status suggests that LPNs and nurses in a full‐time position were at a higher risk of physical and emotional violence compared with their RN colleagues and those in part‐time or casual positions.

### 4.2 Research question 3

Table 5 shows the results of the mediation analyses. Patient/family complaints mediated the relationship between all workload factors and both types of workplace violence. With the exception of unit‐level patient acuity, the relationship between all workload factors and physical violence was partially mediated by patient/family complaints (Sobel tests range: 2.82–4.99). Patient/family complaints fully mediated the relationship between patient acuity and physical violence. Patient/family complaints partially mediated the relationship between all but one of the workload factors and emotional violence (Sobel tests range: 2.90–5.90). The exception was the relationship between patient‐RN ratios and emotional violence that was fully mediated by patient/family complaints.

**Table 5:** Sobel test results for mediation effect of complaints on the relationship between workload factors and physical and emotional violence (*N* = 528)

	a (*SE*)	b (*SE*)	c (*SE*)	Sobel test statistics (*SE*)
Physical violence				
Patient‐RN	0.07*** (0.02)	0.29*** (0.04)	0.05** (0.02)	3.6342*** (0.0055)
Patient acuity^a^	0.53** (0.18)	0.29*** (0.04)	0.42** (0.16)	2.8215** (0.0549)
Heavy workload	0.30*** (0.05)	0.27*** (0.04)	0.26*** (0.05)	4.2724*** (0.0188)
Undone tasks	0.18*** (0.02)	0.24*** (0.04)	0.14*** (0.02)	4.9860*** (0.0089)
Compromised standards	0.24*** (0.04)	0.24*** (0.04)	0.24*** (0.03)	4.6783*** (0.0125)
Interruptions	0.49*** (0.06)	0.24*** (0.04)	0.40*** (0.06)	4.9154*** (0.0232)
Emotional violence				
Patient‐RN^a^	0.07*** (0.02)	0.41*** (0.04)	0.04* (0.02)	3.8183*** (0.0075)
Patient acuity	0.53** (0.18)	0.40*** (0.04)	0.74*** (0.17)	2.8977** (0.0729)
Heavy workload	0.30*** (0.05)	0.36*** (0.04)	0.40*** (0.05)	4.7401*** (0.0227)
Undone tasks	0.18*** (0.02)	0.36*** (0.04)	0.15*** (0.02)	5.9000*** 0.0112
Compromised standards	0.24*** (0.04)	0.34*** (0.04)	0.30*** (0.04)	5.3268*** (0.0155)
Interruptions	0.49*** (0.06)	0.34*** (0.04)	0.50 (0.06)	5.8474*** (0.0281)

Note

a = unstandardized coefficient between workload factors and complaints, b = unstandardized coefficient between complaints and workplace violence when controlling for workload factors, c = unstandardized coefficient between workload predictors and workplace violence, *SE* = standard error

a Full mediation effect of complaints.

* *p* < .05.

** *p* < .01.

*** *p* < .001.

## 5 DISCUSSION

This study had several key findings. First, based on a human factors framework, workload factors at multiple levels were directly related to workplace violence. At the unit level, patient acuity was directly related to increased nurses' reports of emotional violence. At the job level and task level, compromised standards and interruptions, respectively, were directly related to increased reports of both physical and emotional violence. The framework consists of distinct, operational measures for each systems level: Each level has a different impact on nurse outcomes. Unique information about workload impact at different levels can be used to guide approaches to policy change, education and systems design (Holden et al., 2011). Although human factors research is investigating influences of one system level on another (e.g. additive effects), this is a new area of study (Karsh, Waterson, & Holden, 2014).

A second key finding was that patient/family complaints were directly related to increased reports of both types of workplace violence, but there was a stronger relationship with emotional violence than physical violence According to Bandura’s (1991) theory of moral thought and action, through socialization and prevailing social norms and practices, individuals internalize and follow moral standards: they refrain from behaviours that are considered antisocial, such as emotional and physical violence. We postulate that physical violence is more extreme than emotional violence: more external stressors or chronic stress exposure may be required to break social norms and physically aggress against others. Does continued exposure to frustration, anxiety, pain and distress give individuals permission to be violent? How do social norms, as conveyed through organizational antiviolence policies and communications, influence patients' moral and social sense of right from wrong?

Finally, all workload factors at multiple levels were indirectly related to higher reports of physical and emotional workplace violence through the mechanism of patient/family complaints. These findings indicate that patient/family complaints may be a proxy for potential violence towards nurses. In other words, systematic tracking and investigation of patient/family complaints can be used as a workplace violence prevention strategy to warn nursing providers of individuals who may be higher risk for violence. While most research investigated the benefits of tracking complaints for quality and safety of patient care (Gallagher & Mazor, 2015; Pichert, Hickson, & Moore, 2008), there is a dearth of evidence related to its impact on nurse safety.

Two mid‐range theories, in particular, shed light on study findings: stress theory (Russ‐Eft, 2001; Staal, 2004) and spiral of incivility theory (Andersson & Pearson, 1999; Sommovigo, Setti, Argentero, & O’Shea, 2019). According to stress theory, too much stress and/or chronic stress (distress) has detrimental effects on cognition, attention and memory, which in turn adversely influence performance (Russ‐Eft, 2001; Staal, 2004). Heavy workload is a source of distress to nurses, hindering their ability to provide effective patient care. Nursing evidence has established a link between heavy workload, job distress and poor performance (Kokoroko & Sanda, 2019; Li et al., 2017). We surmise that poor quality performance (due to workload factors) is negatively perceived by patients/families who initially respond with complaints. According to the spiral of incivility theory, there is a spiralling process in response to uncivil acts which typically starts with little misbehaviours that can escalate to more serious acts of aggression (Andersson & Pearson, 1999; Sommovigo et al., 2019). If patients/families perceive lack of quality care (e.g. lack of response to call lights) as an uncivil act towards them, this theory suggests that ongoing nursing care concerns will spiral up from complaints to actual acts of emotional and physical violence.

This spiralling process may be triggered when patients' coping strategies are suboptimal due to external causes (e.g. illness, pain) that put them in vulnerable situations (Sommovigo et al., 2019). A systematic review of 53 studies of service providers across a range of industries and sectors used this theory to explain how poor employee performance spirals into client aggression (Sommovigo et al., 2019). Among customer service providers outside of healthcare, poor employee performance was a precursor to client dissatisfaction and incivility such as making gestures (e.g. eye rolling) to express their impatience (Sliter & Jones, 2016). Future research should explore this theory in the context of nurse–patient relationships.

Use of the human factors framework provides a more granular examination of workload factors at different levels that precipitate patient complaints and workplace violence. In this study, certain nurse workload factors, such as job‐level compromised professional standards and task‐level interruptions, were a greater source of distress to nurses than unit‐level and other job‐level workload factors. Rodney (2017) noted that heavy workload was a precursor to moral distress when nurses were unable to uphold their sense of professional integrity and their code of ethics. A Swiss study demonstrated that interruptions to workflow were distressing to nurses. Work interruptions had a negative impact on nurses' attention and concentration, as demonstrated through their failure to actively engage with patients (Pereira, Mueller, & Elfering, 2015).

Patient/family complaints were the strongest predictor of both physical and emotional violence, more so than workload factors. This finding may be because nurse workload is only one source of patient/family complaints. Other factors in the healthcare context such as unmet expectations of patients and families, ineffective communication, lack of resources, wait times and poor care coordination are also known to result in patient dissatisfaction and complaints (Gallagher & Mazor, 2015; Lee, Moriarty, Borgstrom, & Horwitz, 2010; Najafi, Fallahi‐Khoshknab, Ahmadi, Dalvandi, & Rahgozar, 2018). Evidence suggests that patient complaints are often dismissed by healthcare providers as attributions of patient personalities (Gallagher & Mazor, 2015). Patient complaints may be a precursor to emotional and physical violence: They may be the ‘canary in the coal mine.’

### 5.1 Limitations

A key strength of this study was its systematic conceptualization of workload using a human factors framework. Additionally, to our knowledge, this is the first study to examine the mediating effect of patient/family complaints on the relationship between nurse workload factors and workplace violence. A limitation of this study is the difference in the time dimension of the some of the study measures. For example, interruptions to workflow were measured over the last month, but workplace violence and patient/family complaints were measured over the last year. This inconsistency may be a source of bias in the study. Study measures that asked about nurses' experiences over the last year are prone to recall bias. Moreover, nurse reports of workplace violence and patient complaints may not necessarily reflect patient experiences (Duxbury & Whittington, 2005). Future research should triangulate evidence from nurses, patients and their families to examine the impact of nurse workload on complaints and aggressive behaviour. The response rate for the study subsample is unknown but a comparison of the larger study sample with the Canadian Institute of Health Information supported the sample representativeness of the national nursing workforce (blinded). That said, we caution readers against generalizing the findings beyond the study sample and its context. Also, no cause and effect can be established due to the cross‐sectional design of the study. Future research should use more sophisticated research designs (e.g. longitudinal) to examine a cause and effect spiralling process from heavy nurse workload to patient complaints and different types of violence (i.e. emotional, physical).

## 6 CONCLUSION

This study's findings support the importance of using a multisystems human factors framework to examine those workload factors at unit, job and task levels that are associated with workplace violence. The study findings have implications for nurses, managers and policymakers. Heavy workload may be one of the root causes of patient/family violence towards nurses. Employers and policymakers must implement system‐level specific strategies that alleviate nurse workload factors at multiple levels. While most efforts in healthcare target nurse staffing levels as the only unit‐level indicator of heavy workload, new efforts must target workload factors including and beyond nurse–patient ratios. New workload management approaches, for example, advocate for assignments that create a match between patients' care needs and nursing competencies and level of experience (Georgiou, Amenudzie, Ho, & O’Sullivan, 2018).

Our findings also suggest patients and their families respond adversely to nurses' inability to provide effective care due to heavy workloads. Thus, it is important that nurses and managers take patient/family complaints about nursing care seriously. Future research should examine the impact of tracking patient/family complaints on patient and nurse outcomes. According to Gallagher and Mazor (2015), “like any adverse event, patient complaints have an epidemiology that can yield important lessons for prevention ….In many situations, patients and family members may be the first to detect lapses in safety or quality” (p. 352–353). Accordingly, it is important that nurses and managers apply a root cause analysis approach with patient/family complaints as they do with other patient adverse events in health care.

## CONFLICT OF INTEREST

None to declare.

## AUTHOR CONTRIBUTIONS

Havaei has made substantial contribution to this study's conception and design, analysis and interpretation of the data. MacPhee has made substantial contribution to the larger study design and acquisition of the data. Both authors have been involved in drafting the manuscript and revisiting it critically for important intellectual content.

## PATIENT CONSENT

Not required.
